# True congenital dislocation of shoulder: A case report and review of the literature

**DOI:** 10.4103/0973-6042.79798

**Published:** 2010

**Authors:** Pebam Sudesh, Sushil Rangdal, Kamal Bali, Vishal Kumar, Nitesh Gahlot, Sandeep Patel

**Affiliations:** Department of Orthopaedics, Post Graduate Institute of Medical Education and Research (PGIMER), Chandigarh, Punjab, India

**Keywords:** Brachial plexus injury, congenital shoulder dislocation, glenoid hypoplasia, open reduction

## Abstract

The dislocation of a shoulder joint in infancy is extremely rare and is usually the result of traumatic birth injuries, a sequel to brachial plexus injury, or a true congenital dislocation of shoulder. With more advanced obstetric care, the incidence of first two types has drastically decreased. We report a case of true congenital dislocation of shoulder, second of its kind, in a child who was delivered by cesarean section thereby negating any influence of trauma. We report the case because of its rarity, and review the available literature on this topic. We also discuss the management options when encountered with such a rare case scenario.

## INTRODUCTION

“Congenital shoulder dislocation” in true sense is the dislocation of shoulder present at birth, and should logically include cases with dislocation present in utero and those developing during the birth process as a result of trauma. It should not include those cases where the dislocation is secondary to brachial plexus injury and develops gradually due to muscle imbalance. However the classification suggested by Whitmann[1] is commonly quoted and recognizes three types of congenital shoulder dislocations: (a) true congenital dislocation, developing in utero, (b) dislocation caused directly by trauma at birth, and (c) acquired dislocation developing secondary to a brachial plexus injury. The one associated with the brachial plexus injury happens to be the most common type. Delayed presentation of the child only adds to the diagnostic dilemma as to whether the dislocation was actually present at birth.

We present a rare case of a true congenital dislocation of the shoulder in a child. We discuss in detail the diagnostic dilemma in such a case and the management protocol to ensure a good long-term outcome.

## CASE REPORT

A 26-year-old primigravida with an uneventful prenatal course delivered a male child at full term by elective Cesarean section. The indication of the Cesarean section was a transverse lie. APGAR scores and the birth weight were within normal limits. Following delivery, it was noted that movements of the left shoulder in the infant were restricted; however, a complete neurological examination did not reveal any evidence of the brachial plexus injury. The child was initially treated by observation and a gentle range of motion and was referred to our hospital which happens to be a tertiary center for advanced care. He was first seen by us at 4.5 months of age. The mother admitted decreased movements of the left shoulder since child’s birth with inability of the upper limb to be brought to the side of the body.

**Figure 1:**
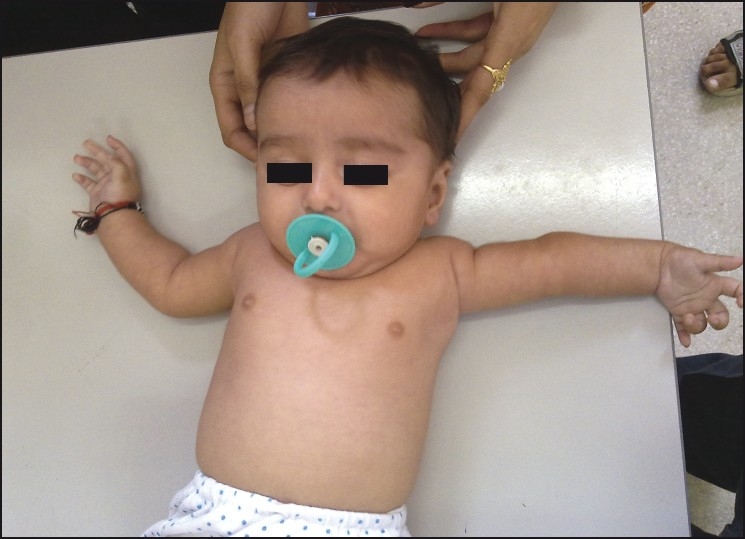
Clinical picture of the infant showing abduction deformity at the left shoulder

A physical examination revealed posturing of the left shoulder in 80° of abduction, neutral flexion, and extension and 30° internal rotation [Figure 1]. Adduction was not possible. There was tightness of the deltoid muscle which probably accounted for the abduction deformity. Passive and active movements at the shoulder were predominantly scapulothoracic. The remainder of the examination of the upper extremity revealed normal findings.

**Figure 2:**
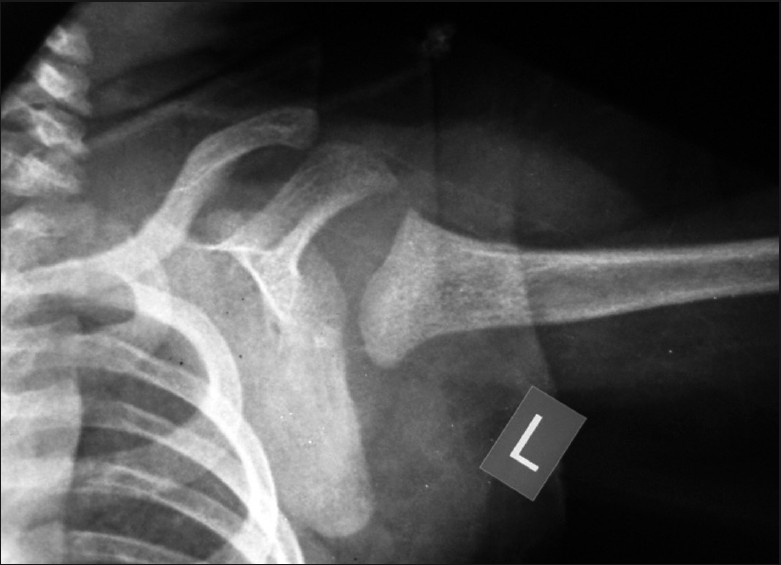
X-ray picture of the left shoulder showing an anteroinferior dislocation

**Figure 3:**
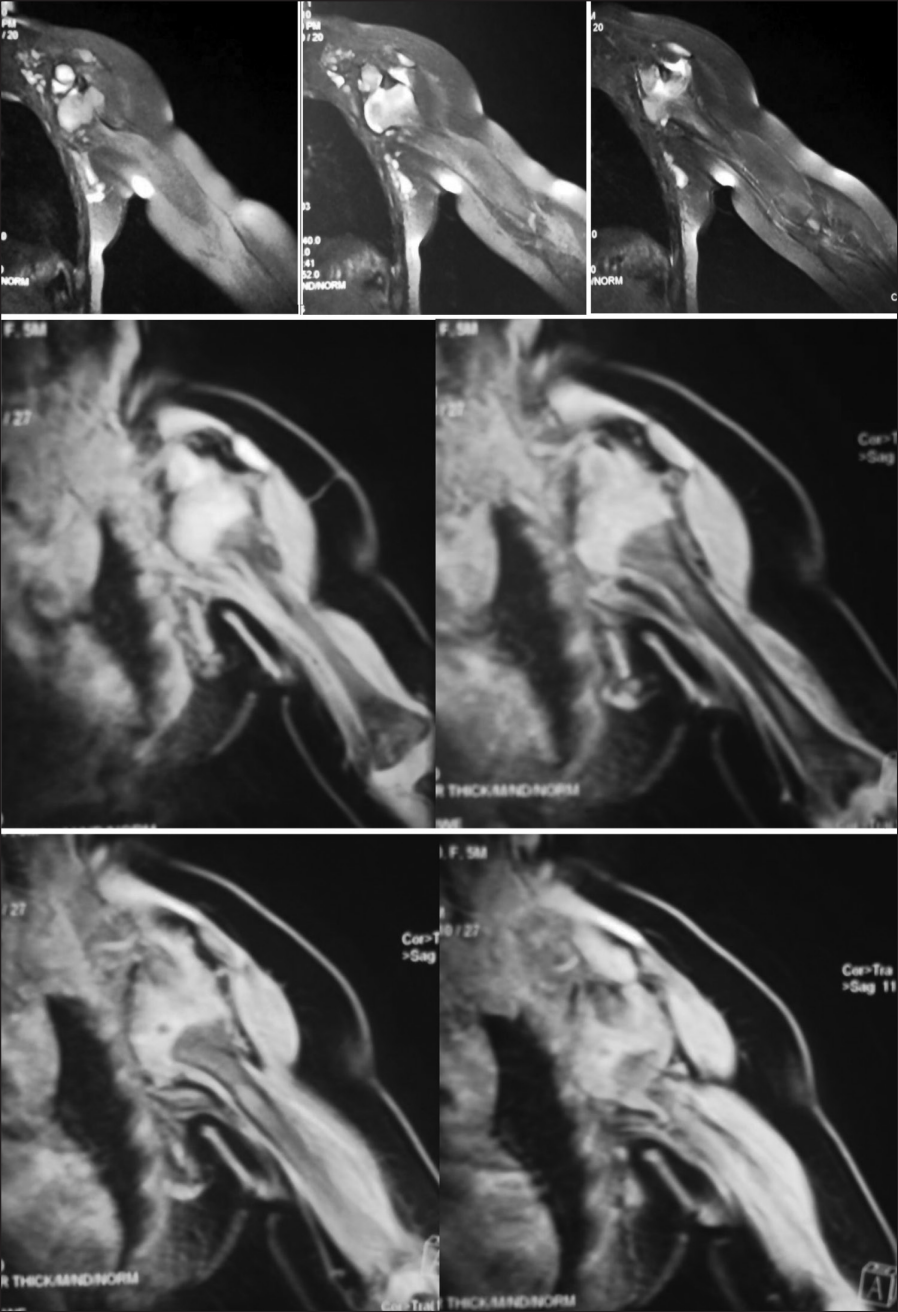
MRI images of the left shoulder showing an anteroinferior dislocation

A plain AP radiograph revealed an anteroinferior dislocation of humeral head with an absent physis [Figure 2]. An MRI scan of the left shoulder [Figure 3] confirmed the above-mentioned findings and also demonstrated a hypoplastic scapula.

**Figure 4:**
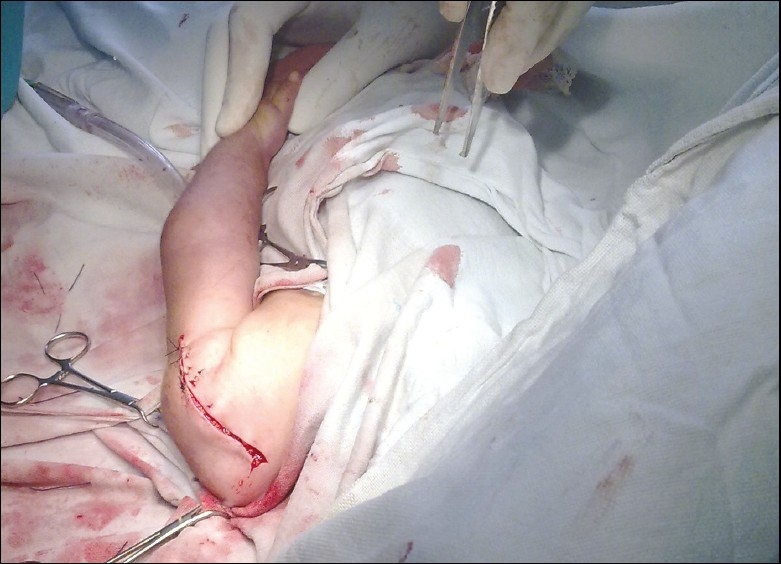
Immediate postoperative clinical picture showing the restoration of adduction in the child

A tight contracted deltoid muscle was preventing reduction by closed manipulation under general anesthesia and therefore open reduction by the standard deltopectoral approach was carried out. The age of the child at that time was around 6 months. Intraoperative findings included anteroinferior dislocation of the head along with scarring of the deltoid muscle which probably accounted for its tightness. A proximal release of the deltoid muscle along with the subscapularis release was done. The inferior joint capsule was surprisingly found to be thickened and tight, and required excision in order to achieve reduction. The glenoid was slightly shallow but there were no other bony abnormalities. Once reduction was achieved, the shoulder was found to adduct fully [Figure 4]. However, the reduction was found to be slightly unstable with the joint being dislocatable at 60° of abduction. A shoulder spica was applied postoperatively in 30° abduction, neutral flexion, and neutral rotation.

The postoperative course was uneventful. Spica removal was done at 6 weeks and passive shoulder movements were started subsequently. At the last follow-up at the age of 1.5 years, the child was able to actively flex shoulder to 100° with a maximum abduction of 90 and an external rotation of 25°. The child is able to keep the upper limb by the side of the body. However, active overhead abduction is still not possible and deltoid muscle stimulation and physiotherapy are being continued.

## DISCUSSION

Stimson[2] was the first to describe the posterior dislocation of the shoulder in infancy associated with brachial plexus injuries in three cases in 1888. Phleps[3] later described three patients who had a congenital posterior dislocation secondary to glenoidal factures (as evident from his intraoperative findings). The isolated dislocation of shoulder without a fracture caused by birth trauma is a rare phenomenon whose existence should be questioned as there are no convincing cases reported so far in the literature. The case reported by Kuhn[4] in reality happens to be a true congenital dislocation and not traumatic as described by him. A true congenital dislocation usually has a hypoplastic scapula and associated shoulder girdle abnormalities which results in unstable reductions. Probably this is the reason why reduction was lost even after closed manipulation as reported by Kuhn.[4] Lemperg’s[5] attempts at producing dislocation in stillborns (regardless of the force and method used) were unsuccessful and instead created fractures, epiphyseal separation, and fracture dislocations.

The existence of a true congenital dislocation of shoulder was also challenged in the early 20^th^ century especially by Taylor.[6] The work of Taylor however threw light on the much debatable topic of congenital shoulder dislocations at that time. He established the presence of a brachial plexus injury as a cause of the congenital shoulder dislocation. This concept was further supported and well described by various other authors.[7–9]

Todd[10] in 1922 through his extensive work in dissections and skeletal survey discussed the possibility of a true congenital dislocation of shoulder and accounted it to the bony abnormalities in the scapula and acromion. Scudder’s[11] description of a case in 1890 is probably the first described case of a true congenital dislocation of shoulder followed by Peckham.[12] The possibility of a true congenital dislocation of shoulder was not considered by these authors and the cases were loosely labelled as a traumatic dislocation of birth. Heilbronner[13] in 1990 presented the first case of the congenital dislocation of shoulder in an infant born by Cesarean section, thus negating the role of trauma.[13] Our case is the second such case in the literature where a congenital dislocation of shoulder can be suspected without a doubt as the our patient was also delivered by Cesarean section. Tables 1 and 2, respectively, summarize the cases of true congenital dislocations and dislocations associated with birth trauma/obstetric palsy. With improved obstetric care and liberal use of Cesarean section in difficult labor, the incidence of congenital shoulder dislocations has drastically decreased in the present era. There have been no fresh cases in the past decade, and the last published case series[14] was a retrospective study including cases from 1967 onward.

**Table 1:** Dislocations of the shoulder associated with obstetrical palsy

Year	Author	Number of cases
1888	Stimson[2]	3
1953	Liebolt *et al*.[7]	2
1955	Wickstrom *et al*.[8]	5
1968	Babbit *et al*.[9]	1
1977	Lichtblau[16]	1
1980	May VR Jr[17]	1
1989	Dunkerton[18]	4
1993	Troum *et al*.[19]	2
2005	Schmelzer-Schmied *et al*.[14]	3

**Table 2:** True congenital dislocation of the shoulder

Year	Author	Number of cases	Mode of delivery
1890	Scudder[11]	1	Vaginal delivery
1905	Peckham[12]	2	Vaginal delivery
1929	Kelly[20]	1	Vaginal delivery
1937	Cozen[21]	1	Vaginal delivery
1984	Kuhn *et al*.[4]	1	Vaginal delivery
1990	Heilbronner[13]	1	Cesarean section
2005	Schmelzer-Schmied *et al*.[14]	4	Vaginal delivery

The differential diagnosis to be considered is the traumatic physeal fracture of the proximal humerus associated with a posterior dislocation, described by Haliburton *et al*.[15] as pseudodislocation and proximal humerus fractures which could be wrongly diagnosed as a true congenital dislocation.[5] CT and MRI are much more sensitive in the diagnosis than radiographs and should be used when in doubt.[22] Ultrasound may also prove to be helpful.

In utero maldevelopment is the major cause for true congenital dislocations of shoulder. This usually occurs due to bony abnormalities of the shoulder girdle.[10–12] In our case, there was hypoplasia of the scapula as depicted in the X-rays and the MRI scans. Glenoid dysplasia as a separate entity has not been described in standard texts, but its presence was pointed by Moukoko *et al*.[23] in 2004 during their study on 134 cases of posterior dislocations of shoulder in children. They theorized that glenoid dysplasia is similar to acetabulum dysplasia as seen in congenital dislocation of the hip and develops due to loss of contact between articular surfaces. Glenoid dysplasia starts improving once the humerus head is relocated in the joint.[24–26]

We seem to disagree with the hypothesis of Heilbronner[13] who attributes the dislocation to abnormal in utero positioning. Abduction of the arm with its rotation against the face is a common attitude of intra utero fetuses and does not increase the likelihood of shoulder dislocations. The contracture of the deltoid muscle is also a contributing factor toward inferior dislocations. Whether the contracture in our case was primary or resulted secondary to the dislocation still remains to be answered.

Early approaches to treatment[3] included resection of the humeral head and closed reduction under anesthesia. However, poor results with such treatments encouraged Fairbank[27] to describe open reduction by an anterior approach where the subscapularis was released and the anterior part of the capsule cut open. A posterior approach was used by Dunkerton[18] while Troum[19] used a combination of anterior and posterior approaches. Wickstrom[8] suggested using the Steinmann pin for securing reduction.

We believe that the principles of treatment are similar to that of the congenital dislocation of hip. Early reduction and containment of the humeral head in the glenoid socket should be the aim. Adequate remodeling with time might help achieve a satisfactory long-term outcome. Open reduction is better as it gives an opportunity to release the contractures and plicate the capsule when necessary and has a lesser chance of redislocation. Our case had milder glenoidal dysplasia and did not require additional K-wire fixation. In higher grades of dysplasia, maintaining reduction with K-wires should be considered. Postoperative spica application in the stable position should be chosen for 6 weeks followed by progressive physiotherapy to regain full shoulder movements.

To conclude, a true congenital dislocation of shoulder is extremely rare. Nevertheless, it should be considered as a possible diagnosis if there is no definitive history of trauma or brachial plexus injury. It is usually the result of bony abnormalities of the shoulder girdle especially that of the glenoid. Timely diagnosis and prompt treatment aimed at reduction, and containment is essential for good results.
